# The Prevalence of Intestinal Helminths in Free-Ranging Canids of Mazandaran, Northern Iran

**Published:** 2019

**Authors:** Abolghasem SIYADATPANAH, Shirzad GHOLAMI, Ahmad DARYANI, Shahabeddin SARVI, Mehdi SHARIF, Mauricio SEGUEL, Larson BOUNDENGA, Afsaneh AMOUEI, Abdol Sattar PAGHEH, Mohammad Taghi RAHIMI, Seyed Abdollah HOSSEINI, Davood ANVARI

**Affiliations:** 1. Ferdows Paramedical School, Birjand University of Medical Sciences, Birjand, Iran; 2. Student Research Committee, Department of Parasitology, Mazandaran University of Medical Sciences, Sari, Iran; 3. Department of Parasitology, Toxoplasmosis Research Center, Mazandaran University of Medical Sciences, Sari, Iran; 4. Odum School of Ecology, University of Georgia, Athens, GA, USA; 5. Department of Medical Parasitology, Group Evolution and Interspecies Transmission of Parasites, International Centre for Medical Research, Franceville (CIRMF), Franceville, Gabon; 6. Department of Parasitology, School of Medicine, Shahroud University of Medical Sciences, Shahroud, Iran; 7. School of Medicine, Iranshahr University of Medical Sciences, Iranshahr, Iran

**Keywords:** Canids, Intestinal helminth, Iran

## Abstract

**Background:**

The purpose of this study was to investigate the current knowledge on the epidemiology of importance zoonotic parasitic diseases in free-ranging canids of Mazandaran, north of Iran.

**Methods:**

Overall, 63 small intestinal samples of animals (20 stray dogs and 43 golden jackals) were collected from April 2017 to May 2018. The intestine contents were studied to detect and identify helminth infections. Additionally, 274 fecal samples (130 dogs, 35 fox, 90 golden jackal and 19 wolf) were examined by Sheather's flotation method for detection of *Taenia* eggs.

**Results:**

Sixty (95.2%) animals were infected with at least one species of intestinal helminth. the intestinal helminths were found in dogs and golden jackals included: *Dipylidium caninum* (25.3%), *Uncinaria stenocephala* (52.3%), *Ancylostoma caninum* (41.2%), *Mesocestoides* spp. (33.3%) and *Toxocara canis* (14.2%). In fecal examination, 2.5% of samples contained *Taenia* eggs, and through a species-specific PCR, 1.09% of these samples were confirmed positive for *Echinococcus granulosus*.

**Conclusion:**

There is a high prevalence and clear risks of zoonotic helminths in free-ranging carnivores in Mazandaran province, north of Iran. Therefore, understanding the epidemiology of zoonotic parasite infection is useful for health care access both domestic animals and humans health.

## Introduction

Free-ranging canids (e.g. dogs, foxes, jackals, wolves), play a significant role as reservoirs of parasitic helminthes for human and livestocks (1–3). There are over 700 million dogs in the world, and approximately 75% of these populations belong to dogs without owner or “strays”. The most important zoonotic diseases transmitted by these groups of carnivorous including echinococcosis, toxocariasis and ancylostomiasis (1, 4, 5). The infective biological forms of these parasites (egg and larvae) are excreted through the feces of the wild and domestic canids. Humans and other animals are accidentally infected through contaminated water and food or penetration of skin by the infective larvae stages from the soil (6). The destruction of the environment of wild animals by humans, climate change and lack of food supplies in their environment has caused them to enter the city's margins and the contact of these animals with humans (7). In some parts of Iran, particularly in the north, where a large population of red foxes, golden jackals and wolves live near the human residence, caused increasing the likelihood of parasite transmission (8). Despite continuous health education and disease prevention programs, and worldwide improvement of cultural and social indicators, soil and food transmitted parasitic zoonoses remain as one of the major health problems in developing countries, causing substantial financial losses every year (9–11).

In Bangladesh, China and Slovenia over 90% of examined canids had intestinal nematodes (12). In Iraq 77.4% (13) and in the USA 33.6% (14) of canids infected by intestinal helminths. Also, in some provinces of Iran such as Chaharmahal and Bakhtiari (15), Khorasan Razavi (16), Mazandaran (17) and Kohgiluyeh and Boyer-Ahmad (18), 71.4%, 84%, 90% and 92% of wild canids contaminated by intestinal helminths, respectively.

The specific climate conditions of Mazandaran Province, with mild average annual temperature and over 800 mm of annual precipitation, provides a perfect condition for the development and transmission of important zoonotic helminths such as hookworms and ascarids (19). Furthermore, because of its location on the southern coast of the Caspian Sea, the Mazandaran Province is one of the most important and popular touristic locations in Iran with thousands of visitors each year. Therefore, the risk of direct contact with soil and water in this area is considerable. All these factors could favor the transmission of helminths from wild canids to humans. However, the parasite species harbored by free-ranging canids, and their prevalence is currently unknown in this region of Iran.

Therefore, the present study aimed to investigate the current knowledge in the epidemiology of parasitic infections and clarifying zoonotic importance of them in free-ranging canids of Mazandaran Province. Moreover, the current survey was designed to understanding the epidemiology of these parasitic infections, which are significant subjects for public health and animal health especially for developing new strategies for treatment and control of zoonotic disease in the north of Iran.

## Materials and Methods

### The study area

A cross-sectional study was conducted from April 2017 to May 2018, in Mazandaran province, north of Iran. This province is located along the southeast coast of the Caspian Sea between 36.2262° N, 52.5319° E and covers an area of 23,842 km^2^. Mazandaran province is geographically separated into the coastal plains and the mountainous areas of Alborz Mountains range. The area has a temperate climate with 70%–100% relative humidity. The average annual temperature and rainfall ranges from 10–35 °C and 800–1200mm, respectively. Mazandaran Province contain various ecosystems including grasslands, sea and forests. This province consists of 15 counties and has a population of around 2,922,432 inhabitants who live in rural and urban areas (17).

### Sampling

**Fig.1:**
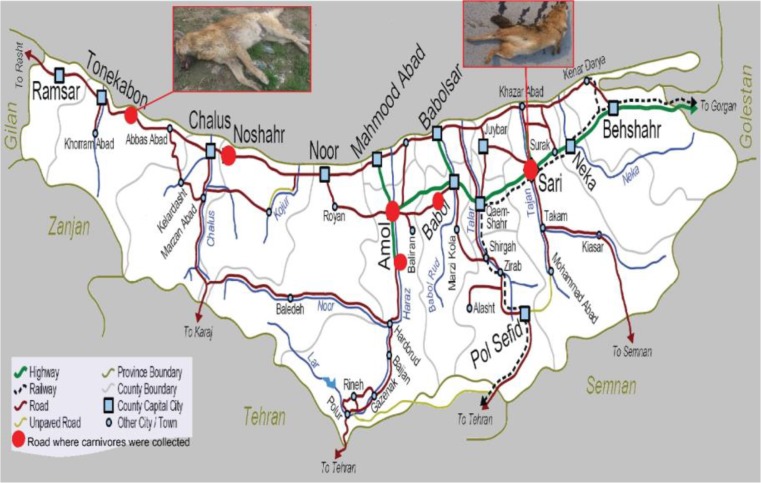
Map of sample collection locations

From April 2017 to May 2018, 20 road-killed stray dogs (*Canis familiaris*) and 43 road-killed golden jackals (*Canis aureus*) carcasses were collected from East, West and Center roads of Mazandaran province (Fig. 1). Additionally, 274 fecal samples from free-ranging canids, including 130 stray dogs, 35 red foxes (*Vulpes vulpes*), 19 wolves (*Canis lupus*) and 90 golden jackals were collected from the three geographical areas (East, Center and West) of Mazandaran province, north of Iran. The identification of feces of different canids’ species can be done by examination of size, shape, contents, and smell (20).

### Parasitological necropsy procedures

The small intestine was cut into 20 cm and sliced longitudinally. Then were placed into a 1 L glass bottle containing normal saline solution *(0.9*% *NaCl)*. In the following, scraping the mucosa from the underlying tissue with a spatula was done. After shaking for approximately 30 min and sediment formation, the supernatant was discarded and the bottle was refilled with normal saline.

This washing procedure was repeated until a clear supernatant was obtained entire sediment was examined in Petri dishes using a stereo microscope (21). Recovered helminths were placed in separate Petri dishes, fixed and preserved. Nematodes were put in glycerin alcohol for clearing; cestodes were pressed gently between the two glass slides and fixed in hot formalin and stained with aqueous carmine and mounted for identification. Identification of intestinal helminths was done based on diagnostic key described in previous studies (22, 23).

### Fecal examination

Fecal samples were examined for detection helminths eggs using the centrifugation-flotation method (24). Briefly, approximately 2 to 3 gr of feces were mixed thoroughly with 10 mL premade Sheather solution (specific gravity 1.27) and transferred to a 15-mL conical tube. In the next step, Sheather solution was added to bring the volume up to 15 mL if required, then, a coverslip (20 mm×20mm) was placed on the tube and the solution was centrifuged at 1200 rpm (280 × *g*) for 5 min. The coverslip was removed, placed on a glass slide and screened at 100 x magnification by light microscopy. Also, six fields were spot-checked using the 40 x magnification. To distinguish and differentiate eggs from each other, the measurement method was used.

### DNA extraction and molecular analysis

The DNA of positive samples for *Taenia* spp. eggs in microscopic examination were extracted using the fast DNA stool Mini *kit WizPrep™ gDNA Mini Kit (Cell/Tissue)* according to the manufacturer’s protocol with slight modifications. All DNA extracts were stored at –20 ºC for use in polymerase chain reaction (PCR). The primers JB3 (5′-TTTTTTGGGCATCCTGAGGTTTAT-3′) and JB4/5 (5′-TAAAGAAAGAACATAATGAAAATG-3′) of the mitochondrial COX1 gene (25) were selected for PCR assay for detection *Echinococcus granulosus*. The PCR reaction was performed 40-μl reaction mixture containing 8 μl of master mix, 25.6 μl of deoxynucleotides (dNTPs), 2.4 μl of primers and 4 μl of DNA template. The PCR program consisted of an initial denaturation step at 94 °C for 5 min, followed by 32 cycles of denaturation at 94 °C for 45 sec, annealing at 55 °C for 45 sec, and extension at 72 °C for 45 sec, and a final extension step at 72 °C for 10 min. The PCR products were analyzed by 1.5% agarose gel electrophoresis and visualized in the UV illuminator.

### Ethical issues

This project was approved by the relevant Ethics Committee of Mazandaran University of Medical Sciences, Sari, Iran.

### Statistical analysis

The results was entered into SPSS ver. 18 (Chicago, IL, USA) and the data were analyzed by Chi-square and Fisher’s exact tests. The confidence level of 95% and *P value* < 0.05 were considered as significant level.

## Results

### Necropsy

**Fig. 2:**
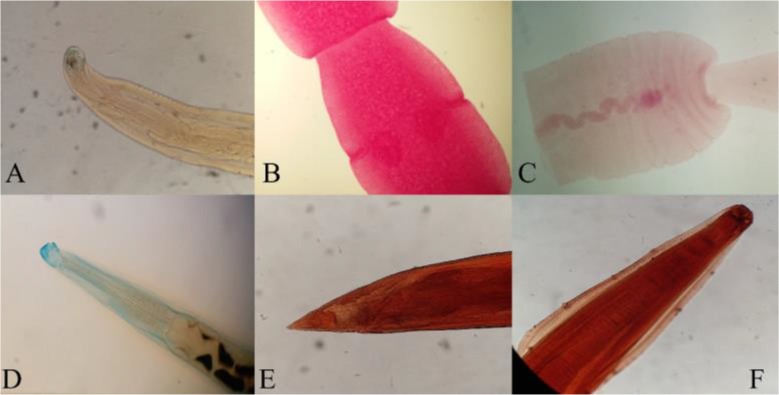
Figures of the intestinal helminths were identified in stray dogs and golden jackals. A: *Ancylostoma caninum* from golden jackal X10, B: Mature proglottid of *Dipylidium caninum* from dog X4, C: Mature proglottid of *Mesocestoides* spp from golden jackal X4, D: *Uncinaria stenocephala* from golden jackal X10, E: Posterior end of *Toxocara canis* from dog X10, F: Anterior end of *T. canis* from dog X10

Overall, 63 free-ranging canids (20 stray dogs and 43 golden jackal) were examined. Sixty (95.2%) of the necropsied animals were infected with at least one helminth species (Table 1 and Table 2). The prevalence of the identified helminths was as follows: *Dipylidium caninum* 25.3%, *Uncinaria stenocephala* 52.3%, *Ancylostoma caninum* 41.3%, *Mesocestoides* spp. 33.3%, *Toxocara canis* 14.2% (Table 1) (Fig. 2). Single infections were identified in 18 (28.5%) of the necropsied canids whereas poly infections were observed in 29 (46.1%) of canids (Table 2). *D. caninum* was the most common helminth parasite in west of Mazandaran (58.3%), whereas *U. stenocephala* was more prevalent in the East (54.1%) and Center (51.8%) of Mazandaran Province (Table 3). Prevalence of intestinal helminths based on gender in dogs and golden jackals was presented in Table 4.

**Table 1:** Prevalence of intestinal helminths in dogs and golden jackals of Mazandaran, northern Iran

***Helminth species***		***Dog No=20 Prevalence (%)***	***Jackals No=43 Prevalence (%)***	***X^2^Fisher’s exact test***	***Total No=63 Prevalence (%)***
Cestoda					
	*Dipylidium caninum*	9 (45)	7 (16.2)	*P*= 0.02	16 (25.3)
	*Mesocestoides* spp*.*	3 (15)	18 (41.8)	*P*=0.03	21 (33.3)
Nematoda					
	*Toxocara canis*	7 (35)	2 (4.5)	*P*=0.03	9 (14.2)
	*Ancylostoma caninum*	8 (40)	18 (41.8)	*P*=0.89	26 (41.2)
	*Uncinaria stenocephala*	6 (30)	27 (62.7)	*P*=0.01	33 (52.3)

**Table 2:** None, mono, poly and intestinal helminthes in dogs and golden jackal examined

***Number of different helminthes***	***Dogs (n=20)***		***Golden jackals (n=43)***		***Dogs and golden jackals (n=63)***	
	***No.***	***(%)***	***No.***	***(%)***	***No.***	***(%)***
Mono infection	8	(40)	10	(21.1)	18	28.5
Double infection	10	(40)	3	(40)	13	20.6
Poly infection	2	(74.4)	27	(37.8)	29	46.1
Total infection	20	(100)	40	(93.1)	60	95.2

**Table 3:** Prevalence of intestinal helminths in dogs and golden jackals in 3 locations of Mazandaran, northern Iran

***Helminth species***		***West (No= 12) Prevalence (%)***	***Jackals and dogs Center (No=27) Prevalence (%)***	***East (No= 24) Prevalence (%)***	***X^2^d.f=2***
Cestoda					
	*Dipylidium caninum*	7 (58.3)	5 (18.5)	4 (16.6)	8.6: P=0.01
	*Mesocestoïdes spp*	2 (16.6)	10 (37.1)	9 (37.5)	1.8: P=0.39
Nematoda					
	*Toxocara canis*	1 (8.3)	3 (11.1)	5 (20.8)	1.4: P=0.49
	*Ancylostoma caninum*	5 (41.6)	10 (37.1)	11 (45.8)	0.41: P=0.81
	*Uncinaria stenocephala*	6 (50)	14 (51.8)	13 (54.1)	0.06: P=0.97

**Table 4:** Prevalence of intestinal helminths in male and female dogs and golden jackals from northern Iran

***Helminth species***		***Male No= 24 Prevalence (%)***	***Female No=39 Prevalence (%)***	***X^2^Fisher’s exact test***
Cestoda				
	*Dipylidium caninum*	4 (16.6)	12 (30.7)	*P*=0.22
	*Mesocestoïdes spp*	9 (37.5)	12 (30.7)	*P* =59
Nematoda				
	*Toxocara canis*	6 (25)	3 (7.6)	*P* =0.07
	*Ancylostoma caninum*	10 (41.6)	16 (41.1)	*P* =0.95
	*Uncinaria stenocephala*	11 (45.8)	22 (56.4)	*P* =0.42

### Fecal Examination

Taeniid eggs were observed in 7 (2.5%) of 274 total fecal samples, by microscopic examination. *E. granulosus* was identified by PCR in 1 out of the 7 samples positive for Taeniid eggs through microscopic examination.

## Discussion

Transmission of certain helminth parasites of wild canids to domestic animals and humans, particularly in rural areas, causes many economic and public health problems (26). Approximately 60% of human infections are zoonotic and are transmitted from animals to humans. A considerable proportion of these pathogens are helminthic parasites that cause important diseases in humans and animals (27).

Our results showed that the overall prevalence of helminth parasites in wild canids was 95.2%. This finding is similar to other parts of Iran such as Moghan Plain (96%) and Mashhad (90%) (2, 28). In this study, *U. stenocephala* was the most prevalent helminth with a prevalence of 62.7% in golden jackals and 30.0% in dogs. A similar prevalence has been reported in wolves (66.6%) from Italy (29). Moreover, several researchers from Iran and other countries have reported this nematode as the most common helminth in canids (2, 26, 29, 30). One of the reasons for this regard could be due to the wet and warm climate of Mazandaran, which provides optimal conditions for the life cycle of this parasite (19).

*A. caninum,* was the second most frequently recorded nematode in current study. This hookworm can cause eosinophilic enteritis and chronic abdominal pain in human (31, 32). Prevalence of *A. caninum* in the present study was 41.2% in dogs and golden jackals. This finding is similar to other studies conducted in dogs from other countries such as Malaysia (48%) (33) and Mexico (42.9%) (34).

Results of necropsy in wild canids showed that, *Mesocestoides* spp. was the most common Cestoda (33.3%). This finding is similar to the prevalence of this helminthic infection in red foxes (*V. vulpes*) in Portugal (30.7%) (35). Whereas, in contrast to our results, prevalence of *Mesocestoides* spp. was 84.7% and 73.2% in moghan plain of Iran (2) and Greece (36), respectively. One of the important reasons for the high prevalence of this parasite is the presence of a large number of the intermediate hosts (e.g. arthropod, small mammals, birds, reptiles and amphibians) in the life cycle of this parasite, which increases the chances of survival and transmission of the parasite to the wild carnivores (15).

*D. caninum* is considered a common intestinal parasite in canids (37, 38). The prevalence of this parasite in our study was 25.3%, which considered low compared to previous studies conducted in other parts of Iran such as Mashhad (39%) (28) and Tabriz (52.5%) (39). Moreover, high prevalence of *D. caninum* has been observed in dogs from Nigeria, with a prevalence of 75% (40). Dipylidiasis is a neglected parasitic zoonosis caused by *D. caninum*, a common intestinal tapeworm of dogs and cats. Dogs are infected by ingestion of the flea or louse containing the cysticercoids (41). In this regard, due to the high probability that stray dogs are contaminated with fleas that carriers of this parasite, the high prevalence of *D. caninum* in these dogs can be justified.

*T. canis* was the less common nematode parasite in current study with a prevalence of 14.2%. This is comparable to other studies in Iran (16.0%) (42), Egypt (26.6%) (43) and Tunisia (27.2%) (44). *T. canis* is common parasite in the canids and causes toxocariasis which is one of the major zoonotic diseases transmitted by the infected animals (45). Humans are accidental hosts who may become infected by ingesting embryonated eggs through contaminated vegetables, fruits and water or by direct contact with infected carnivores. Human infection may cause intense damage generally to the eye, the liver, and the lungs (46, 47). Thus, their abundance in the environment could constitute a veritable health problem for the human population exposed to this nematode worm.

According to WHO reports, more than a million people are infected with hydatid cyst in the world (48). This zoonotic disease is caused by the larval form of the tapeworm *Echinococcus* spp. In life-cycle of *E. granulosus*, dogs and wild carnivores act as the final host and ruminants are usual intermediate host. Humans are accidental hosts via directly contact with final hosts or indirectly through with ingestion water, vegetable or soil contaminated by infective eggs. In spite of, many efforts for control of the zoonotic parasitic diseases, cystic echinococcosis remains a dangerous public health problem in Iran (8, 26, 49).

Based on fecal examination and PCR survey one sample was positive for *E. granulosus*. This finding is concurrent with previous studies in Hamedan province (50, 51). Similar to our previous study, we have not found *Echinococcus* spp. cestodes in the intestine of the necropsied canids, probably due to the low prevalence of this parasite in the studied canid population. In the other regions of Iran, the incidence rates of *E. granulosus* in canids have been reported from 3.0% to 50.5% (17). Many factors affect the prevalence of this helminth in canids, including management of intermediate host viscera at rural slaughterhouses, feeding practices of pets and stray dogs and the treatment of final and intermediate hosts.

## Conclusion

There are still the clear risks of zoonotic helminths parasites infection in free-ranging canids in Mazandaran Province, Iran. The high prevalence of zoonotic parasitic diseases supposes a risk for public and environmental health and suitable operational methods should be taken to regulate the populations of free-ranging canids such as stray dogs, to prevent transmission of these infectious diseases. Therefore, epidemiological studies should be conducted regularly and seasonally throughout the country, especially in high-risk areas. Moreover, health education programs and control of the parasitic infections could be reduced the transmission of canine intestinal parasitic infections in the north of Iran.
